# Feasibility of MR-Based Body Composition Analysis in Large Scale Population Studies

**DOI:** 10.1371/journal.pone.0163332

**Published:** 2016-09-23

**Authors:** Janne West, Olof Dahlqvist Leinhard, Thobias Romu, Rory Collins, Steve Garratt, Jimmy D. Bell, Magnus Borga, Louise Thomas

**Affiliations:** 1 Department of Medical and Health Sciences, Linköping University, Linköping, Sweden; 2 Center for Medical Image Science and Visualization (CMIV), Linköping University, Linköping, Sweden; 3 Advanced MR Analytics AB, Linköping, Sweden; 4 Department of Biomedical Engineering, Linköping University, Linköping, Sweden; 5 Nuffield Department of Population Health, University of Oxford, Oxford, United Kingdom; 6 UK Biobank, Stockport, United Kingdom; 7 Research Centre for Optimal Health, Department of Life Sciences, Faculty of Science and Technology, University of Westminster, London, United Kingdom; INIA, SPAIN

## Abstract

**Introduction:**

Quantitative and accurate measurements of fat and muscle in the body are important for prevention and diagnosis of diseases related to obesity and muscle degeneration. Manually segmenting muscle and fat compartments in MR body-images is laborious and time-consuming, hindering implementation in large cohorts. In the present study, the feasibility and success-rate of a Dixon-based MR scan followed by an intensity-normalised, non-rigid, multi-atlas based segmentation was investigated in a cohort of 3,000 subjects.

**Materials and Methods:**

3,000 participants in the in-depth phenotyping arm of the UK Biobank imaging study underwent a comprehensive MR examination. All subjects were scanned using a 1.5 T MR-scanner with the dual-echo Dixon Vibe protocol, covering neck to knees. Subjects were scanned with six slabs in supine position, without localizer. Automated body composition analysis was performed using the AMRA Profiler^™^ system, to segment and quantify visceral adipose tissue (VAT), abdominal subcutaneous adipose tissue (ASAT) and thigh muscles. Technical quality assurance was performed and a standard set of acceptance/rejection criteria was established. Descriptive statistics were calculated for all volume measurements and quality assurance metrics.

**Results:**

Of the 3,000 subjects, 2,995 (99.83%) were analysable for body fat, 2,828 (94.27%) were analysable when body fat and one thigh was included, and 2,775 (92.50%) were fully analysable for body fat and both thigh muscles. Reasons for not being able to analyse datasets were mainly due to missing slabs in the acquisition, or patient positioned so that large parts of the volume was outside of the field-of-view.

**Discussion and Conclusions:**

In conclusion, this study showed that the rapid UK Biobank MR-protocol was well tolerated by most subjects and sufficiently robust to achieve very high success-rate for body composition analysis. This research has been conducted using the UK Biobank Resource.

## Introduction

Two of the greatest health-challenges today are the increasing prevalence of obesity and the risks associated with aging. Obesity is, amongst others, closely associated with type-2 diabetes [1, 2], cardio-vascular diseases [2–5], neurovascular disease [4], and some types of cancers [2], resulting in increased mortality and decreased quality of life. Similarly, sarcopenia, the loss of muscle mass observed with aging or following trauma or osteoarthritis [6–9], is strongly associated with decreased quality of life and increased disability [10, 11]. Other conditions that are associated with local or general decrease in muscle mass include muscular dystrophies [12, 13], spinal cord injuries [14], and sport injuries [15].

Quantitative and accurate measurements of body fat and muscle are therefore important for the prevention and diagnosis of diseases related to obesity and sarcopenia. In population-based studies, the association between body composition and other biomarkers as well as disease progression are of interest. Several methods have been put forward as potential tools to determine body composition in small and large cohorts, including dual-energy x-ray absorptiometry (DXA) [16] and bioimpedance (BIA) [17]. However, these methods do not allow direct quantification of absolute compartmental tissue volumes in a consistent and accurate manner.

Magnetic resonance imaging (MRI), currently the gold standard for body composition analysis, allows for accurate quantification of body fat content and distribution and skeletal muscle mass. Furthermore, water-fat separated MRI, based on Dixon imaging techniques [18] enables high soft-tissue contrast and the separation of fat and muscle compartments. Scanning the whole body with sufficient resolution to separate muscle and fat compartments may be accomplished in less than ten minutes, and for neck-to-knee coverage in a mere six minutes. However, it may be challenging to maintain high throughput and data acquisition quality as the number of subjects increases and the time-per-scan decreases. In particular, it is challenging to implement an effective and robust MRI acquisition and analysis protocol without operator intervention, such as using a localizer or the need of highly specialised personnel.

Manual and/or semi-automated methods have been the principal approach for quantification of muscle and fat compartments from MR images. However, these methods are often laborious and introduce significant intra- and inter-operator variability. Furthermore, while they may be acceptable for smaller cohorts, they quickly become unmanageable as the number of subjects increase. Because of this, the implementation of automated quantification methods for large-scale studies has been of increasing interest recently.

Methods that automatically identify and quantify muscle and fat compartments are either based on whole-body segmentation [19–23] or limited to specific parts of the body [24–26], and quantify body fat [19, 20, 26] or body muscle [21–25]. The combined fat and muscle quantification is not commonly reported.

In previous studies, cohorts have been relatively limited, such as 80 subjects in [27], 477 subjects in [28], and 314 in [19]. Recently, however, there has been increasing interest in advanced phenotyping in ever-larger cohorts. In particular, the UK Biobank imaging study [29] will include 100,000 subjects who undergo radiological examinations, including neck-to-knee body MRI. In The German National Cohort [30], 30,000 subjects will undergo radiological examinations, including whole-body MRI. In order to handle these immense data quantities it is crucial to develop methods for rapid, accurate and robust body composition analysis.

The aims of this study were to **(1)** investigate the feasibility and success-rate of one recently described method for MR data-acquisition and body composition analysis, in a large-scale population study, **(2)** define a standard set of criteria for acceptance/rejection of datasets and **(3)** assess the capability of an automated image analysis system for such cohort.

## Materials and Methods

### Overview

Data acquisition with a Dixon-based MR-protocol was performed in the first 3,000 subjects of the UK Biobank multimodal-imaging cohort. Analysability and a number of technical quality assurance metrics were investigated for the initial 1,000 datasets. Based on the technical quality assurance a reduced data-acceptance protocol was developed. The protocol was subsequently applied on the following 2,000 subjects. Finally, visceral adipose tissue (VAT), abdominal subcutaneous adipose tissue (ASAT), and thigh muscles volumes were quantified for the accepted subjects.

### In-vivo acquisition

3,000 subjects from the UK Biobank multimodal-imaging cohort were included in this prospective study. The MR acquisitions were part of a more extensive scanning protocol, including neuro-imaging, cardiac-imaging, and DXA-scan of bones and joints. For full details, see [29]. The age range for inclusion was 40–69 years of age, and subjects were excluded if they had metal or electrical implants, had surgery within six weeks before scanning, or if they had medical conditions that would make it difficult to conduct the scans, such as severe hearing or breathing problems. While this feasibility study was performed, the researchers were blinded as to the demographics and other results to the study cohort. The inclusion rate was 20 subjects/week during the first two weeks. This rate was subsequently increased until, in week 16, the full inclusion rate of 126 subjects/week was reached.

All subjects were scanned using a Siemens Aera 1.5 T scanner (Syngo MR D13) (Siemens, Erlangen, Germany) with the dual-echo Dixon Vibe protocol, covering neck to knees. Subjects were scanned in supine position with the arms along the sides, the MR-landmark was positioned on the subject’s clavicles and no localizer was used. The Dixon protocol covered a total of 1.1 m divided over six overlapping slabs of axial 3D spoiled gradient dual-echo images. Reconstruction of water-fat Dixon images was performed using the integrated scanner software. Common parameters for all slabs were; TR = 6.69 ms, TE = 2.39/4.77 ms, and bandwidth 440 Hz. The first slab, over the neck consisted of 64 slices, voxel size 2.23×2.23x3 mm^3^ and 224×168 matrix; slabs two to four were acquired during 17 sec expiration breath-holds with 44 slices, voxel size 2.23×2.23×4.5 mm^3^ and 224×174 matrix; slab five consisted of 72 slices, voxel size 2.23×2.23×3.5 mm^3^ and 224×162 matrix; slab six consisted of 64 slices, voxel size 2.23×2.23×4 mm^3^ and 224×156 matrix. The North West Multicenter Research Ethics Committee (MREC), UK, approved the study and written informed consent was obtained from all subjects prior to study entry.

### Technical Quality Assurance

Technical quality assurance was performed and reported for the first 1,000 subjects in order to evaluate prevalence of artifacts and to investigate factors affecting analysability. Five analysis engineers, all employed by AMRA (Advanced MR Analytics, Linköping, Sweden) and trained to visually assess radiological images, performed the quality assurance using an extensive questionnaire. The analysis engineers considered a set of image-artifacts and attributes as defined below. Once all datasets had been inspected, the questionnaires were subsequently compared to each subject once again by one operator (J.W.) to ensure consistency. In cases of disagreement, a discussion was held to reach consensus. The following aspects were included in the technical quality assurance protocol:

#### Analysability

Analysability for VAT, ASAT and thigh muscle was assessed. All reasons for compartments deemed as not analysable were noted. Compartments were considered not analysable if any part of the anatomical region was missing in the dataset.

#### Respiratory artifacts in image

Respiratory artifacts in an image were defined as visible motion artifacts over the abdominal region. This was present when the subject was not able to hold their breath over a complete breath-hold acquisition.

#### Metal contamination

Metal contamination was defined as areas of distinct signal-voids in the MR-signal. Metal implants were an exclusion criterion for this study. Nonetheless, in the extensive group of subjects, this may have been missed in some cases, or the subject may not always be aware of metal filings.

#### Water-fat swaps

When the image reconstruction was performed, the algorithm needed to determine the signal-channel for fat and water. In cases where this was ambiguous the algorithm may have interchanged the two channels, leading to water-compartments being detected in the fat-image and fat-compartments being detected in the water-image. This is commonly known as water-fat swaps. The most common water-fat swap is a separate-island swap, where a complete region disconnected from surrounding tissue is associated with the wrong signal-channel, *e*.*g*. one thigh or the complete abdomen. One special case of separate-island swap is when the top of the liver is wrongly detected as fat. Water-fat swaps may also occur inside of other regions although this is less common. In the inspection-questionnaire, four types of water-fat swaps were included: Water-fat swaps in subcutaneous adipose tissue (SAT) (excluding swaps due to outer field-of-view (FOV) inhomogeneities, which are reported separately), water-fat swaps in top of liver, water-fat swaps in thigh, and separate-island swaps (excluding swaps in top of liver, which are reported separately). Swaps in top of liver were subsequently corrected by excluding the swapped liver-top in the VAT segmentation. Other separate-island swaps were corrected by interchanging the water and fat signal channels before segmentation.

#### Outer FOV inhomogeneities

Outer FOV inhomogeneities were defined as water-fat swaps that occurred close to the edges of the FOV, where the MR-signal diminishes due to drops in the main magnetic field. This type of water-fat swap visually resembles bites and is therefore sometimes termed *“dog-bites”*.

#### Incomplete knee coverage

In this study, a neck-to-knee protocol was used, spanning a fixed length from the subject's clavicles. Hence, in subjects who were either very tall, or placed too low in the MR scanner, the lower parts of the legs may have been outside the scanned volume. The knees were considered complete if all of the femoral epicondyles were visible in the MR images.

### Data-Acceptance Protocol

A reduced data-acceptance protocol, with a standard set of criteria for acceptance/rejection of datasets, was established based on the technical quality assurance. The factors that determined the analysability were identified and these were included as basis for acceptance or rejection of each independent measurement. The subsequent 2,000 subjects were investigated using the data-acceptance protocol.

### Body composition analysis

Body composition analyses for all approved datasets were performed using the method previously described in [22, 31, 32]. Briefly, the analysis consisted of the following steps:

1. The fat and water image volumes were intensity inhomogeneity corrected and calibrated using the algorithm described in [31] and [33]. This method uses pure adipose tissue as an internal signal reference. The images were subsequently merged into a composite set of fat and water image volumes covering the neck to the knees.
2. Atlases with ground truth labels for fat compartments: visceral adipose tissue (VAT), and abdominal subcutaneous tissue (ASAT), as well as muscle compartments: left posterior thigh, right posterior thigh, left anterior thigh, and right anterior thigh were registered to the acquired volumes using non-rigid atlas based registration, as described in [22]. VAT was defined as the adipose tissue within the abdominal cavity, excluding adipose tissue outside the abdominal skeletal muscles and adipose tissue and lipids within and posterior of the spine and posterior of the back muscles. ASAT was defined as subcutaneous adipose tissue in the abdomen from the top of the femoral head to the top of the thoracic vertebrae T9. Posterior thigh muscles were defined as gluteus, iliacus, adductor and hamstring muscles on respective sides and anterior thigh muscles were defined as quadriceps femoris and sartorius. The atlas database consisted of up to 31 prototypes of both genders. The prototypes were selected from previously segmented datasets, encompassing a wide variety of phenotypes in the UK Biobank imaging study. Selection was made in two steps: First, histograms of VAT and ASAT were genereated in order to find candidates to be included in the atlas. Then, a large number of candidates were visually inspected to find suitable prototypes. All ground truth atlases were inspected and approved by a trained analysis engineer prior to being used in this study.
3. Quantification of fat and muscle volumes was performed using a voting scheme based on the registered labels and the intensity corrected fat and water images. Initially, each registered label provided a vote for each label in the target. The target was subsequently segmented by assigning each voxel the corresponding label if more than five atlases agreed.
4. Finally, after the automatic segmentation, all datasets were visually inspected by an analysis engineer. The operator could interactively adjust the final segmentation in subjects with atypical anatomies (*i*.*e*. atypical compared to the atlases). For additional details see [32].

For all acquisitions, volumes of VAT and ASAT were calculated by integrating the calibrated fat-image over the final quality assured labels. Thigh muscle volumes (left anterior, right anterior, left posterior and right anterior) were calculated as fat-free muscle volume using the method described in [22]. All body composition analyses were performed in AMRA Profiler^™^ (AMRA AB, Linköping, Sweden).

### Statistical Analysis

Descriptive statistics were calculated for all volume measurements and quality assurance metrics. Furthermore, histograms were generated for VAT, ASAT, total trunk fat defined as VAT + ASAT and total thigh volumes. All statistical analyses were performed in SPSS 21 (SPSS Inc., Chicago, USA, 2012).

## Results

Of the 3,000 subjects, 2,775 (92.50%) were fully analysable for fat and thigh muscle, 2,828 (94.27%) were analysable when fat and only one thigh was included, and 2,995 (99.83%) were analysable when omitting both thighs.

**Fig 1:**
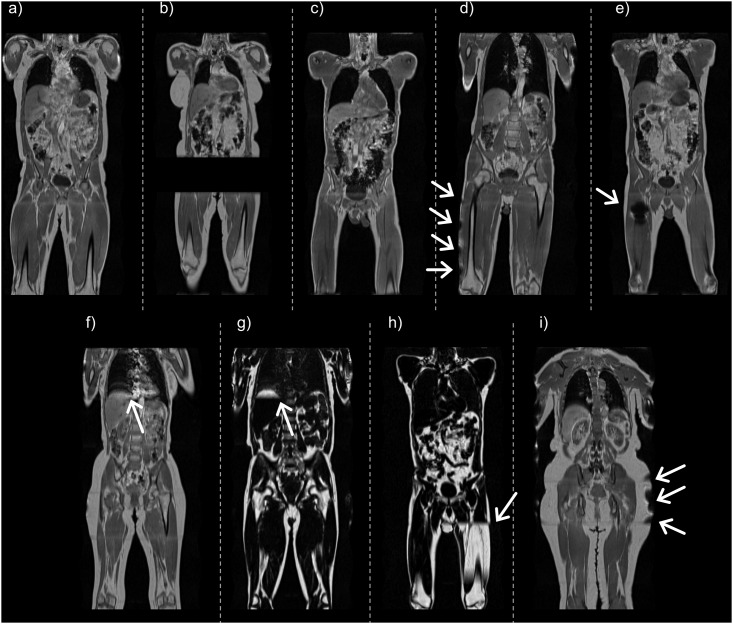
Typical imaging artifacts within the UK Biobank imaging study. (a) tall subject where the complete femoral epicondyles are not visible, thighs are not analysable, (b) missing slab over the abdominal and thigh regions, not analysable, (c) misplaced landmark where the complete femoral epicondyles are not within the imaged volume, thighs are not analysable, (d) tilted subject where the right thigh is partly outside the field-of-view, right thigh not analysable (e) metal artifact, right thigh not analysable, (f) respiratory artifact, visible as ghosting on top of liver, (g) swap in top-of-liver, (h) separate-island swap, this was corrected, and (i) outer field-of-view inhomogeneities. Characteristic artifacts are highlighted with arrows.

In the extensive technical quality assurance of the first 1,000 subjects, outer FOV inhomogeneities were detected in almost one-third of the subjects, and about one-in-ten were not able to hold their breath during the complete acquisition leading to respiratory artifacts in the abdominal region. Metal was detected in three of the subjects. Reasons for not being able to analyse either fat or muscle in the dataset were: either due to missing slabs in the acquisition (12.77%, six subjects), patient positioned so that large parts of the volume was outside of the FOV (82.98%, 39 subjects), metal artifacts causing signal-voids (2.13%, one subject), or in one case due to severe motion-artifacts. Details on the technical quality assurance are reported in Table 1, and representative samples of each type of artifact are demonstrated in Fig 1. The reduced data-acceptance protocol is specified in Table 2, and the details from the subsequent 2,000 subjects are reported in Table 3. In particular, a majority of the images deemed as not analysable fell within the defined criteria, however, 4 subjects were found not to be analysable due to metal artifacts and 23 subjects were too tall to fit within the imaged volume. This was reported as *other*, with an additional note.

**Table 1:** Technical quality assurance of the first 1,000 subjects.

QA measurement	Number of subjects (n = 1,000)	
Analysable VAT + ASAT	997	99.70%
Analysable VAT + ASAT + One thigh	986	98.60%
Analysable VAT + ASAT + Two thighs	981	98.10%
Respiratory artifacts	134	13.40%
Metal contamination	3	0.30%
Water-fat swaps in SAT*	2	0.20%
Water-fat swaps in top of liver	67	6.70%
Water-fat swaps in thigh	13	1.30%
Separate-island swaps	30	3.00%
Outer FOV inhomogeneities	327	32.70%
Incomplete knee coverage	60	6.00%

* Not including outer FOV inhomogeneities, which are reported separately.

**Table 2:** Reduced data-acceptance protocol.

Criteria	Description	Type
VAT and ASAT analysable		Y/N
Reason for no: Missing slabs	Are slabs missing over the abdominal region?	Checkbox
Reason for no: Landmark position invalid	Is the abdominal region completely or partly outside the FOV, in particular is vertebrate T9 present?	Checkbox
Reason for no: Other, specify		Text
Left thigh analysable		Y/N
Right thigh analysable		Y/N
Reason for no: Missing slabs	Are slabs missing over the thigh region?	Checkbox
Reason for no: Landmark position invalid	Is the thigh region completely or partly outside the FOV, in particular is the femoral epicondyles visible?	Checkbox
Reason for no: Tilted subject	Are the subject titled in the scanner so that part of the thigh is outside the FOV, or so that severe outer FOV inhomogeneities affect the thigh muscles?	Checkbox
Reason for no: Other, specify		Text

**Table 3:** Acceptation and rejection of the subsequent 2,000 datasets based on the data-acceptance protocol.

QA measurement	Number of subjects (n = 2,000)	
Analysable VAT + ASAT	1,998	99.90%
• Missing slabs	1	0.50%
• Landmark position invalid	0	0.00%
• Other	1	0.05%
Analysable Left thigh	1,839	91.95%
Analysable Right thigh	1,857	92.85%
• Missing slabs	11	0.55%
• Landmark position invalid	62	3.10%
• Tilted subject	39	1.95%
• Other	32	1.60%

**Fig 2:**
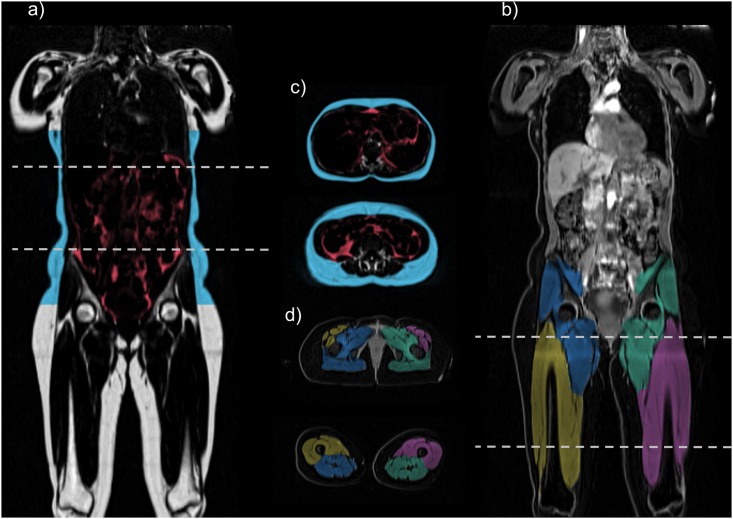
Coronal and transverse slices of one subject. (a) intensity-corrected coronal fat image with fat segmentations using overlay colours, (b) intensity-corrected coronal water image with muscle segmentations using overlay colours, (c) corresponding transverse slices with fat segmentation, and (d) corresponding transverse slices with muscle segmentation.

**Fig 3:**
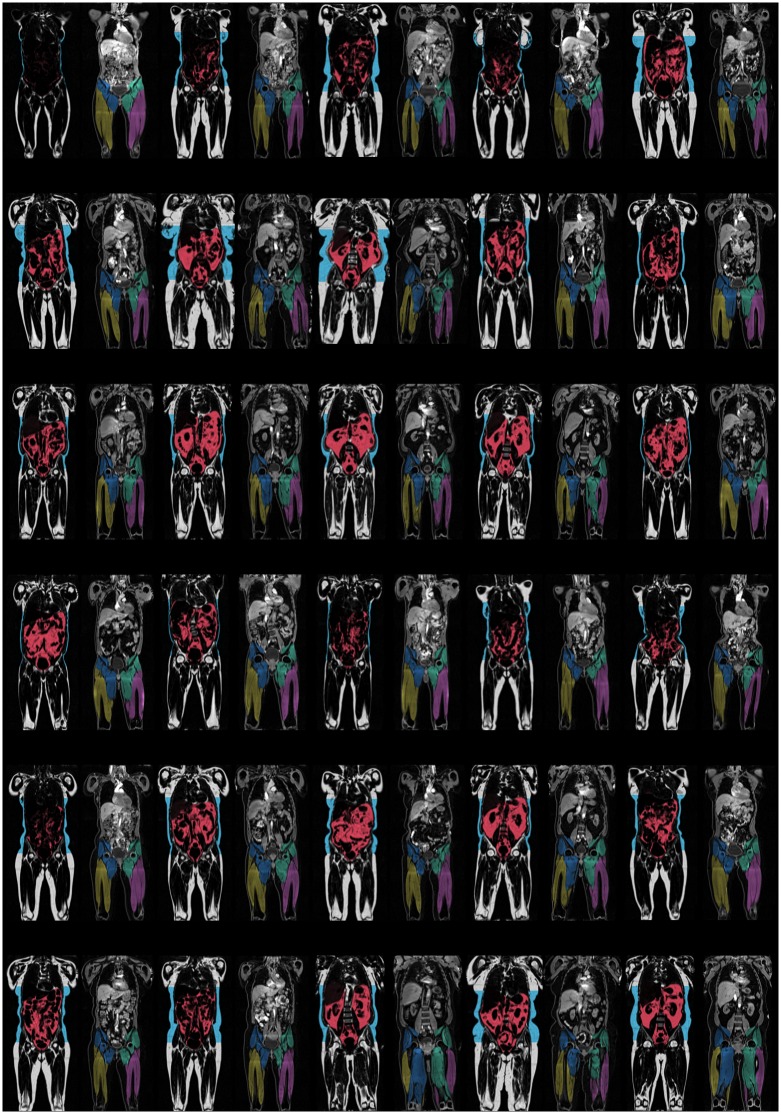
Central coronal slices from 1% of the imaged subjects. Demonstrating the wide range of phenotypes within the UK Biobank imaging study. For each subject; left shows intensity-corrected coronal fat image with fat segmentations using overlay colours, right shows intensity-corrected coronal water image with muscle segmentations using overlay colours.

**Fig 4:**
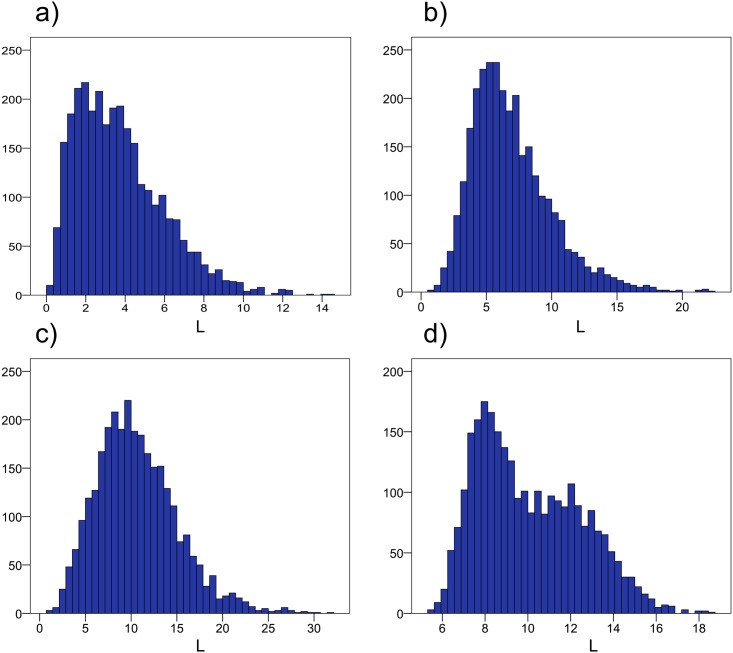
Histograms from all 3,000 subjects. (a) visceral adipose tissue (VAT), (b) abdominal subcutaneous tissue (ASAT), (c) total trunk fat (defined as VAT+ASAT), and (d) total thigh volumes.

Fig 2 shows a representative segmentation from one subject, in both coronal and transversal planes. In Fig 3, a 1%-sample of the total imaged cohort is shown, demonstrating the wide range of phenotypes within the UK Biobank imaging study. The mean VAT volume was 3.73 ± 2.23 L (range 0.10 L to 14.36 L), the mean ASAT volume was 6.91 ± 3.06 L (range 0.65 L to 22.05 L), the mean total trunk fat volume (VAT+ASAT) was 10.64 ± 4.43 L (range 1.00 to 31.63 L) and the mean total thigh volume was 10.06 ± 2.44 L (range 5.34 to 18.49 L). Complete descriptive statistics for the volume measurements are reported in Table 4. Histograms of VAT, ASAT, total trunk fat, and total thigh volumes are shown in Fig 4.

**Table 4:** Descriptive statistics. All 3,000 subjects, subjects with missing data are excluded list-wise.

MRI measurement	mean ± SD
VAT [L]	3.73 ± 2.23
ASAT [L]	6.91 ± 3.06
Total trunk fat [L]	10.64 ± 4.43
Total thigh [L]	10.06 ± 2.44
Right anterior thigh [L]	1.68 ± 0.47
Right posterior thigh [L]	3.39 ± 0.80
Left anterior thigh [L]	1.66 ± 0.45
Left posterior thigh [L]	3.36 ± 0.79

## Discussion

Here we report the outcome of the first 3,000 subjects in the UK Biobank undergoing in-depth phenotyping by means of a customized MR-protocol for body composition analysis. The main aims of this study were to investigate the feasibility and success-rate of rapid MR-based body acquisition and analysis. Furthermore, a standard set of acceptance/rejection criteria was defined and applied. The MR examination was completed within 6-minutes including three 17-seconds breath-holds. The high success-rate indicated that the customized MR-protocol was well tolerated by the volunteers within the UK Biobank, a cohort that closely reflects the general adult population in the UK.

Data acquisition was followed by an off-site analysis using the AMRA Profiler^™^ protocol, as previously described in a smaller cohort [32]. Over 99% of images were determined as analysable (absence of significant artifacts in regions of interest) and therefore were included for body composition analysis. In general, most types of artifacts were infrequently observed, except outer FOV inhomogeneities, which were predominately observed in very large subjects and which may have contributed to a lower sensitivity for the ASAT measurement. For example, the abdominal fat compartments for two subjects were partially outside of the FOV. For these subjects, the ASAT measurement did not include all fat depots, and hence led to lower sensitivity. Water-fat swaps were uncommon, suggesting that the Dixon Vibe reconstruction was very robust. Furthermore, by including quality assurance and strict criteria for data-acceptance, the data was assessed for reliability on a per subject basis, and not only for the whole population. This is an advantage in precision medicine where each individual scan must be of the highest possible quality [34].

Furthermore, the presented body composition analysis method could well be applied in multi-point Dixon imaging (such as 3- or 6-point) which may be clinically benificial *e*.*g*. for the quantification of muscle fat-fraction.

### Limitations

In the UK Biobank study, neck-to-knee coverage was achieved using an MR-protocol with six slabs. While this was sufficient for most volunteers, some scans did not cover the complete thighs because of height of some subjects. Including an additional slab can overcome this, but it would of course prolong the overall examination time by c.a. 60 seconds. Although seemly a relatively short periods of time, in a large population study such as the UK Biobank, where up to 100,000 subjects are participating, an increase of 60 seconds would entail an overall increase in scanning of 69 days. This simple example demonstrates the importance of time-efficiency in large-scale studies.

Finally, although the instruction in the UK Biobank study was to perform a complete rescan if any part of the acquisition failed, some datasets did show missing slabs, probably because of errors in the scanning. This effect was in fact one of the greatest reasons rendering datasets un-analysable. This error can of course be easily overcome by the simple introduction of automated software to inspect and quality control slab acquisition.

## Conclusions

In conclusion, this study showed that the rapid MR body protocol implemented in the UK Biobank was very well tolerated by the subjects and extremely robust to achieve very high success-rate for body composition analysis. This study suggests that the presented method can be readily applied in population-wide studies for precision medicine.

## Supporting Information

S1 File Full UK Biobank acknowledgements. Imaging Working Group and other relevant UK Biobank committees.
(PDF)
