# Supplementary material for: Feasibility of MR-Based Body Composition Analysis in Large Scale Population Studies
Source: PLoS One. 2016 Sep 23;11(9):e0163332. doi: 10.1371/journal.pone.0163332 (PMC5035023; doi:10.1371/journal.pone.0163332)
Supplement: S1 File — Imaging Working Group and other relevant UK Biobank committees. (PDF) [file pone.0163332.s001.pdf]

Imaging Working Group and other relevant UK Biobank committees

Imaging working group membership:

Chair: Prof Paul Matthews
Division of Brain Sciences, Imperial College London

Assoc Prof Naomi Allen
Nuffield Department of Population Health, University of Oxford;
& UK Biobank Senior Epidemiologist

Prof Jimmy Bell
Department of Life Sciences, University of Westminster, London

Prof Andrew Blamire
Newcastle Magnetic Resonance Centre, Newcastle University

Prof Sir Rory Collins
Nuffield Department of Population Health, University of Oxford;
& UK Biobank Principal Investigator

Dr Tony Goldstone
Department of Medicine, Imperial College London

Prof Nicholas Harvey
MRC Lifecourse Epidemiology Unit, University of Southampton

Prof Alan Jackson
The Wolfson Molecular Imaging Centre, University of Manchester

Prof Paul Leeson
Oxford Cardiovascular Clinical Research Facility, University of Oxford

Prof Karla Miller
Oxford Centre for Functional MRI of the Brain, University of Oxford

Prof Stefan Neubauer
Oxford Centre for Clinical Magnetic Resonance Research,
University of Oxford

Prof Steffen Petersen
William Harvey Research Institute, Queen Mary College London

Prof Stephen Smith
Oxford Centre for Functional MRI of the Brain, University of Oxford

Prof Cathie Sudlow
Centre for Clinical Brain Sciences, University of Edinburgh;
& UK Biobank Chief Scientist

Clinical Trial Service Unit & Epidemiological Studies Unit, University of Oxford:

Prof Martin Landray; Dr Alan Young; Dr Michael Lay

UK Biobank Coordinating Centre:
Ms Nicola Doherty (Senior Clinical Study Administrator);
Mr Steven Garratt (Senior Project Manager);
Ms Kirsty Lomas (Programme Manager);
Dr Tim Peakman (Deputy CEO);
Mr Jonathan Sellors (Legal counsel); and
Dr Simon Sheard (Director of Operations)

UK Biobank Imaging Assessment Centre: Ms Sarah Hudson (Lead Radiographer)

University of Edinburgh: Dr Lorna Gibson (Radiology Clinical Research Fellow)

University of Cardiff: Ms Maggie Gregory (Participant Resource Centre Manager)

UK Biobank Executive Team

CEO: Prof Sir Rory Collins (University of Oxford/UK Biobank)

Deputy CEO: Dr Tim Peakman (UK Biobank)

Chief Scientist: Prof Cathie Sudlow (University of Edinburgh/UK Biobank)

Senior Epidemiologist: Assoc Prof Naomi Allen (University of Oxford/UK Biobank)

Legal Counsel: Mr Jonathan Sellors (UK Biobank)

Director of Operations: Mr Simon Sheard (UK Biobank)

Finance Director: Mr Grant Nash (UK Biobank)

Head of Communications: Mr Andrew Trehearne (University of Oxford/UK Biobank)

Senior Study Administrator and Secretariat: Ms Nicola Doherty (UK Biobank)

UK Biobank Steering Committee

Chair: Prof Sir Rory Collins (University of Oxford/UK Biobank)
Assoc Prof Naomi Allen (UK Biobank)
Prof John Danesh (University of Cambridge)
Prof Paul Elliott (Imperial College London)
Prof John Gallacher (University of Oxford)
Prof Jane Green (University of Oxford)
Prof Paul Matthews (Imperial College London)
Dr Tim Peakman (UK Biobank)
Prof Jill Pell (University of Glasgow)
Assoc Prof Tim Sprosen (University of Oxford)
Prof Cathie Sudlow (UK Biobank)
Secretariat: Nicola Doherty (UK Biobank)

UK Biobank Enhancement Working Group

Chair: Prof Paul Elliott (Imperial College London)
Assoc Prof Naomi Allen (UK Biobank);
Prof Sir Rory Collins (UK Biobank / University of Oxford)
Prof Frank Kelly (Kings College London)
Dr Julian Marchesi (Imperial College London and University of Cardiff)
Dr Tim Peakman (UK Biobank)
Prof Naveed Sattar (University of Glasgow)
Dr Augustin Scalbert (IARC, Lyons)
Prof Cathie Sudlow (UK Biobank)
Dr Ioanna Tzoulaki (Imperial College London)
Prof Tony Whetton (University of Manchester)
Secretariat: Nicola Doherty (UK Biobank)

Groups and individuals consulted on imaging project

Brain MRI Advisory Group

Chair: Prof Steve Williams (Centre for Neuroimaging Sciences; London)
Prof John Ashburner (WT Centre for Neuroimaging; London)
Prof Nick Fox (Dementia Research Centre; London)
Prof Paul Matthews (Division of Brain Sciences; Imperial College London)
Prof Karla Miller (Oxford Centre for Functional MRI of the Brain; Oxford)
Prof Tom Nichols (Institute for Digital Healthcare; Warwick)
Prof Stephen Smith (Oxford Centre for Functional MRI of the Brain; Oxford)
Prof David Van Essen (NIH Human Connectome Project; Washington, USA)
Prof Meike Vernooij (Rotterdam Study; Holland)
Prof Henry Volzke (German National Cohort; Germany)
Prof Nikolaus Weiskopf (WT Centre for Neuroimaging; London)

Other brain MRI experts consulted:
Dr Christian Beckmann (Donders, Holland)
Prof Michael Chappell (Oxford)
Dr Larry Clarke (NCI, USA)
Prof Heidi Johansen-Berg (Oxford)
Prof Derek Jones (Cardiff)
Dr Thomas Okell (Oxford)
Dr Sebastien Ourselin (UCL London)
Prof Geoff Parker (Manchester)
Prof Tomas Paus (Nottingham/Rotman)
Dr Jonathan Roiser (UCL London)
Prof Gunter Schuman (King's College London)
Prof Christian Schwarzbauer (SINAPSE/Aberdeen)
Prof Jon Shah (Juelich, Germany)
Dr Kaveh Vahedipour (Helmholtz cohort; Germany)
Prof Joanna Wardlaw (SINAPSE/Edinburgh)
Prof Mike Weiner (California, USA)
Prof Julie Williams (Cardiff)

Cardiovascular MR image analysis consortium

Mr Nicholas Ayache (INRIA)
Mr Herve Delingette (INRIA)
Prof James Duncan (Yale)
Ms Jane Francis (Oxford)
Prof Alex Frangi (University of Sheffield)
Dr A Gooya (University of Sheffield)
Prof Stefan Neubauer (Oxford)

Prof Wiro Nissen (Erasmus MC)
Prof Alison Noble (Oxford)
Prof Sebastian Ourselin (UCL London)
Prof Steffen Petersen (QMUL)
Dr Stefan Piechnik (Oxford)
Prof Daniel Rueckert (Imperial College London)
Dr Graham Wright (Toronto)
Prof Alistair Young (University of Auckland)

Carotid Ultrasound Advisory Group

Chair: Dr Paul Leeson (Oxford Cardiovascular Clinical Research Facility; Oxford)
Mr Ram Bedi (Panasonic)
Mr Takenori Fukomoto (Panasonic)
Mr Rudy Meijer (Panasonic)
China Kadoorie Biobank.

Other cardiovascular disease experts consulted

Prof Harald Becher (Edmonton, Canada)
Prof Robert Clarke (Oxford)
Prof Henry Dargie (Glasgow
Dr Aaron Fenster (Robarts Imaging Research Laboratories; Canada)
Prof Valentin Fuster (Mount Sinai Cardiovascular Institute; USA)
Dr Michael Jerosch-Herold (Boston, USA)
Dr Francisco Leyva (Birmingham)
Dr Gerry McCann (Leicester)
Dr Pieter Muntendam (BGMedicine; USA)
Dr Bernard Prendergast (Oxford)
Prof Jeannette Schulz-Menger (Berlin, Germany)
Prof Henrik Sillesen (Department of Vascular Surgery; Denmark)
Prof David Spence (Stroke Prevention & Atherosclerosis Research Centre; Canada)
Prof Sir Magdi Yacoub (Imperial College London)
Clinical Trial Committee of Society for Cardiovascular Magnetic Resonance (SCMR)
ESC Working Group on Cardiovascular Magnetic Resonance
British Society for Cardiovascular Magnetic Resonance

Body MRI Advisory Group

Chair: Prof Jimmy Bell (University of Westminster; London)
Co-chair: Dr Tony Goldstone (MRC Clinical Sciences Centre; London)
Dr Rajarshi Banerjee (Radcliffe Dept. of Medicine; Oxford)
Prof Magnus Borga (Linkoping University; Sweden)
Prof Fiona Gilbert (Biomedical Imaging Centre; Aberdeen)
Dr. Amy Herlihy (Oxford)
Prof Paul Matthews (Imperial College London)
Prof Matthew Robson (Radcliffe Dept. of Medicine; Oxford)
Prof Robert Ross (School of Kinesiology and Health Studies; Canada)
Prof N Jon Shah (Juelich-Aachen Brain Research Alliance; Germany)
Prof Wei Shen (Columbia University Obesity Research Center; USA)
Dr Lidia Szczepaniak (Cedars Sinai Medical Center; USA)
Dr Garry Tan (Diabetes and Endocrinology Department; Nottingham)
Dr Louise Thomas (University of Westminster; London)
Dr Andoni Toms (Musculosketal Radiology; Norwich)
Prof Henry Volzke (German National Cohort; Germany)
Prof John Waterton (Translational Imaging; Manchester)

Musculoskeletal Advisory Group

Chair: Prof Cyrus Cooper (MRC Lifecourse Epidemiology Unit; Southampton)
Co-chair: Prof Nick Harvey (MRC Lifecourse Epidemiology Unit; Southampton)
Prof Juliet Compston (Bone Medicine; Cambridge)
Prof Richard Eastell (Mellanby Centre for Bone Research; Sheffield)
Prof Roger Francis (Bone Clinic; Newcastle)
Prof Stuart Ralston (Rheumatic Disease Unit; Edinburgh)
Prof David Reid (Institute of Medical Sciences; Aberdeen)
Prof Alan Silman (Botnar Research Centre; Oxford)
Prof Jon Tobias (Academic Rheumatology; Bristol)

Other experts and organisations consulted:
Prof Judith Adams (Manchester)
Prof Tim Cootes (Manchester)
Dr Nicola Crabtree (Birmingham)

Radiology consultants

Prof Judith Adams (Manchester)
Dr Ermanno Capuano (London)
Prof Alan Jackson (Manchester)
Dr Stephen Lee (Manchester)
Dr Alexia Rossi (QMUL)

Dr Francesca Pugliese (William Harvey Research Institute, London)

Organisations/bodies consulted

Academic Committee of the Royal College of Radiology
Association of British Neurologists (London)
Christie Medical Physics (Manchester)
National Institutes of Health (Washington DC; USA)
Society and College of Radiographers
UK Biobank Ethics & Governance Council
